# Preventing eye injuries

**Published:** 2015

**Authors:** Daksha Patel

**Affiliations:** E-learning Director: International Centre for Eye Health, London School of Hygiene and Tropical Medicine, London, UK.

**Figure:**
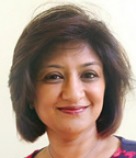
Daksha Patel

The main challenge in developing a strategy to prevent eye injuries is that there are so many different causes and situations that can lead to eye injuries, each requiring a different approach.

In general, the first step in prevention is to inform people about the risks so that they can either avoid them or take action to protect their eyes. People can be informed by means of appropriate safety messages (e.g. on posters) in the areas where eyes are at risk, or through a range of media campaigns. In some environments, such as in industrial settings, messages can be supported by education/training sessions.

**‘In some high-risk environments, information and education is not enough’**

Prevention messages and education certainly increase people's knowledge about avoiding and protecting themselves against risks, but there is insufficient evidence that these, by themselves, will result in changed behaviour in the long term. A recent Cochrane review^2^ indicates that the overall impact of education is short-lived and that it often needs to be reinforced or supported in practical ways. For example, in high-risk environments (e.g. agricultural and industrial settings) where people are advised to wear protective eyewear (safety glasses), it might be necessary to also provide the correct eyewear. This will improve compliance.

When considering the use of protective eyewear, it is important to pay attention to the practicalities: is the eyewear comfortable to use, and is it suited to the task at hand? Eye protection has to be suitable in its visual properties (i.e. can people see well enough?), size and weight and must have the appropriate strength of material for the protection required.

The influence of legislation on risk reduction has been well documented. In the UK, studies have demonstrated a reduction of up to 73% in motor vehicle-related eye injuries after the introduction of compulsory seat belt use.^1^

Injuries in the home lack a specific pattern of aetiology and are therefore the most challenging to prevent. Creating general awareness about the safe use of domestic chemicals, kitchen equipment or gardening tools is therefore essential. Safety standards for toys, tools and home equipment might also be needed in some countries.

**Table 1.** Suggestions for the prevention of eye injuries at individual and community level

	**Prevention at individual level**	**Prevention at community/public health level**
**Home**	Keep sharp objects/chemicals away from children and look for safety standards in household products	Raise safety awareness on the use of tools and kitchenware around the house
**Industry**	Emphasise the use of helmets and eye protection	Raise awareness and advise industries on safer modifications of the work environment. May require introduction of safety legislation
**Agricultural**	Encourage the use of eye protection, particularly at harvest time	Audit injuries and their seasonality so that appropriate advice/education can be provided e.g. to fruit pickers or during grain harvests
**Sport**	Encourage the use of eye protection and/or helmets, e.g. for contact sports and racquet sports	Consider advocating for legislation to encourage compliance with protective eyewear use
**Conflict**	Give advice on the importance of using helmets and protective eyewear	Lobby government to provide protective gear and appropriate training for soldiers
**Assault**	Difficult to advice specific action at individual level	Encourage and support multidisciplinary action to reduce violence at a community level
**Transport**	Encourage motorists to wear seatbelts and cyclists and motorcycle users to wear eye protection	Advocate for legislation to support compliance
**Fireworks**	Promote keeping a safe distance during firework use, especially for children	Organise prevention messages in media during periods of festivity
**Contact lenses**	Give advice on contact lens wearing habits and discourage overnight use	Raise awareness among eye workers on lens types and correct wearing habits for contact lens users

Despite the challenges, prevention is essential. Table 1 gives suggestions for the prevention of the most common eye injuries, both at an individual and at a community/public health level. Overleaf there is a case study on prevention in the agricultural environment and one looking at evidence on eye injury prevention in a work setting. In our online edition, there is also a case study looking at the prevention of road traffic accidents in Kenya by ensuring that commercial drivers have good visual health and good visual acuity (**www.cehjournal.org/article/vision-testing-to-prevent-road-traffic-accidents-in-kenya/**). This will in turn help to prevent eye injuries. A next step would be the introduction of seat belt wearing – backed up by legislation – to prevent an accident from causing blinding injuries.
